# STAT2 R148 variant: A 16th-century founder mutation and clinical response to high-dose JAK inhibitor therapy

**DOI:** 10.70962/jhi.20260001

**Published:** 2026-05-27

**Authors:** Nima Parvaneh, Rasol Molatefi, Conor Gruber, Sajjad Biglari, Leila Moradi, Yoann Seeleuthner, Fatima Ailal, Ahmed Aziz Bousfiha, Neda Pak, Camille Soudée, Jean-Laurent Casanova, Jérémie Rosain, Dusan Bogunovic, Mohammad Reza Fazlollahi, Mohammad Shahrooei, Jacinta Bustamante

**Affiliations:** 1 Division of Allergy and Clinical Immunology, Department of Pediatrics, Children’s Medical Center, Tehran University of Medical Sciences, Tehran, Iran; 2 Cancer Immunology and Immunotherapy Research Center, Ardabil University of Medical Sciences, Ardabil, Iran; 3 Department of Pediatrics, Bo-Ali Children’s Hospital, Ardabil University of Medical Sciences, Ardabil, Iran; 4 Department of Pediatrics, Vagelos College of Physicians and Surgeons, Columbia University Irving Medical Center, New York, NY, USA; 5 Columbia Center for Genetic Errors of Immunity, Vagelos College of Physicians and Surgeons, Columbia University, New York, NY, USA; 6 Division of Rheumatology, Children’s Hospital of Philadelphia, Philadelphia, PA, USA; 7 Farin Genetics Laboratory, Tehran, Iran; 8 Immunology, Asthma and Allergy Research Institute, Tehran University of Medical Sciences, Tehran, Iran; 9 Imagine Institute, Paris Cité University, Paris, France; 10 Laboratory of Human Genetics of Infectious Diseases, INSERM U1163, Necker Hospital for Sick Children, Paris, France; 11 Laboratory of Clinical Immunology, Infection and Autoimmunity, Faculty of Medicine and Pharmacy of Casablanca, Hassan II University, Casablanca, Morocco; 12 Department of Pediatric Infectious Diseases and Clinical Immunology, Children’s Hospital, CHU Averroes, Casablanca, Morocco; 13 Radiology Department, Children’s Medical Center, Tehran University of Medical Sciences, Tehran, Iran; 14 Department of Pediatrics, Necker Hospital for Sick Children, Paris, Assistance Publique-Hôpitaux de Paris, Paris, France; 15 St. Giles Laboratory of Human Genetics of Infectious Diseases, Rockefeller Branch, The Rockefeller University, New York, NY, USA; 16 Howard Hughes Medical Institute, New York, NY, USA; 17 Center for the Study of Primary Immunodeficiencies, Necker Hospital for Sick Children, Assistance Publique-Hôpitaux de Paris, Paris, France; 18 Division of Pediatric Allergy, Immunology and Rheumatology, Department of Pediatrics, Columbia University, New York, NY, USA; 19 Dr. Shahrooei Lab, Tehran, Iran

## Abstract

STAT2 R148 variants cause severe type I interferonopathy by disrupting USP18-mediated negative feedback regulation. We studied two new Iranian patients homozygous for *STAT2* p.R148Q variant presenting with life-threatening neuroinflammation and respiratory failure. Patient 1 developed seizures, brain calcifications, and severe pneumonia, achieving dramatic improvement with high-dose ruxolitinib. Patient 2 presented with lymphadenopathy, encephalitis, and recurrent infections and died from respiratory failure at 8.5 years. Haplotype and principal component analysis (PCA) analysis revealed a founder variant originating ∼491 years ago in the Middle East/North African region. A review of five published cases and these two patients demonstrated constant neurological involvement and a high mortality rate. Early recognition and high-dose JAK inhibitor therapy may improve outcomes in this devastating but potentially treatable interferonopathy.

## Teaser

Patients with STAT2 p.R148Q variant present with life-threatening neuroinflammation, respiratory failure, and high mortality rate. Early genetic recognition and treatment with high-dose JAK inhibitors may improve outcomes in this devastating but potentially treatable interferonopathy.

## Graphical Abstract

**Figure:**
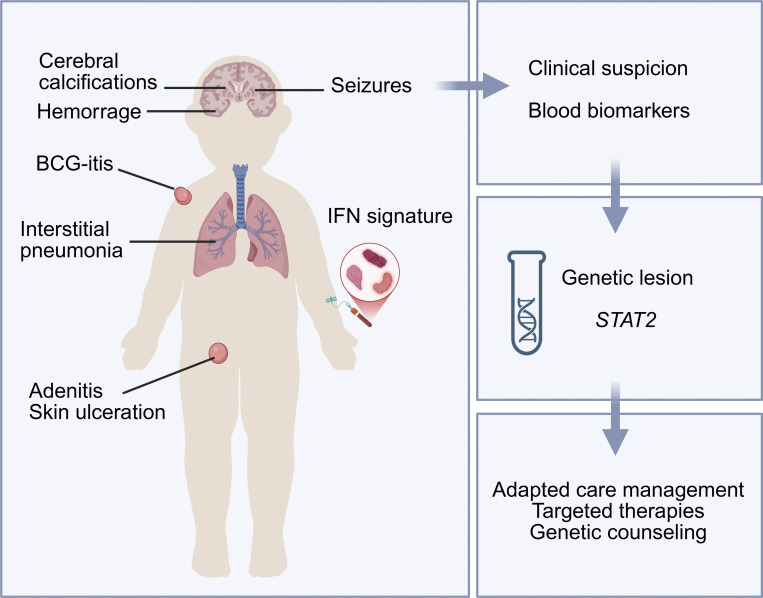

## Introduction

Signal transducer and activator of transcription 2 (STAT2) is a transcription factor crucial for regulating type I and III interferons (IFN). The IFN signaling relies on STAT2, which, together with STAT1 and IFN regulatory factor 9, forms the IFN-stimulated gene (ISG) factor 3 (ISGF3) transcriptional complex. ISGF3 induces the transcription of ISGs and mediates antiviral immunity (1, 2). In humans, pathogenic biallelic *STAT2* variants cause two distinct autosomal recessive (AR) disorders with distinct functional consequences. Loss-of-function (LOF) variants (missense, nonsense, essential splicing site, and small or large deletion) attenuate type I IFN signaling and increase susceptibility to disseminated viral infections, such as encephalitis, influenza pneumonia, or systemic reactions after live attenuated viral measles, mumps, and rubella vaccine (1, 2, 3, 4, 5, 6, 7, 8). Another biallelic missense variants cause constitutive pathway activation, producing type I interferonopathy (9, 10, 11, 12, 13, 14). These variants described either prevent ubiquitin-specific peptidase 18 (USP18) binding (p.R148W, p.A219V) or impair USP18 trafficking to IFN-α/β receptor β chain (IFNAR2) (p.R148Q), resulting in sustained IFN signaling and chronic inflammation. These disorders exhibit a broad clinical spectrum, ranging from neuroinflammation to severe pulmonary disease (9, 12). Although the neurologic prognosis is guarded and mortality rates are high, Janus kinase (JAK) inhibitors have recently emerged as a targeted therapy (13, 14, 15). Here, we report two unrelated Iranian patients with a homozygous *STAT2* p.R148Q variant who presented with severe type I interferonopathy. To place these cases in context, we also reviewed the literature to characterize the clinical spectrum, functional consequences, and therapeutic responses associated with STAT2 R148LOF/regulation.

## Results

### Case reports of two new patients with rare biallelic *STAT2* R148Q variants

#### Patient 1 (P1) (Kindred A)

**Figure 1.**
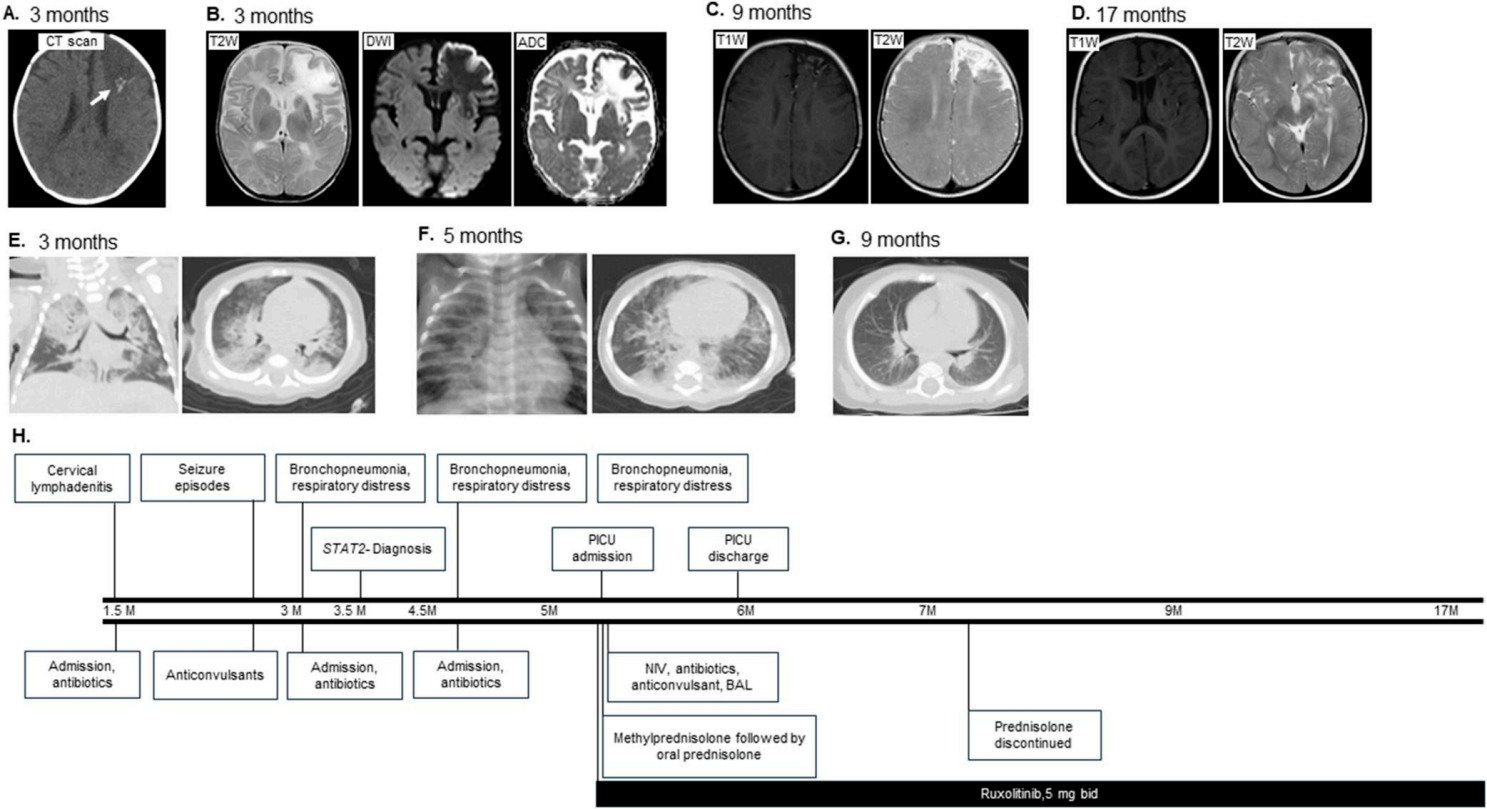
**Clinical course of and treatment response of P1.** Timeline of key clinical events, imaging findings, and therapies from 1.5 to 17 mo of age. **(A)** Intracranial calcifications on brain CT scan (indicated by an arrow). **(B)** Brain MRI at 3 mo of age: T2-weighted image (left), diffusion-weighted imaging (DWI, middle), and apparent diffusion coefficient (ADC, right) showing hypoxic-ischemic encephalopathy with areas of patchy hemorrhage in the left frontal lobe and basal ganglia. **(C)** Brain MRI (T1-weighted and T2-weighted) performed at 9 mo of age showing atrophy and encephalomalacia in the left frontal lobe and left basal ganglia. **(D)** Follow-up brain MRI at 17 mo of age showing no active lesions. **(E)** Thoracic CT showing lung opacities compatible with pneumonia. **(F)** Bilateral alveolar opacities demonstrated on chest x-rays and lung CT scan. **(G)** Peribronchial thickening observed on chest CT scan. **(H)** Both neurologic and pulmonary manifestations improved following initiation of ruxolitinib therapy. Treatment included noninvasive ventilation (NIV), antibiotics, BAL, and high-dose methylprednisolone followed by oral prednisolone. After confirmation of a STAT2 p.R148Q variant, ruxolitinib was initiated. Follow-up showed clinical improvement with stabilization of oxygen saturation, resolution of respiratory symptoms, improvement on lung CT, and absence of active CNS lesions on brain MRI by 17 mo. PICU, Pediatric Intensive Care Unit.

**Figure 2.**
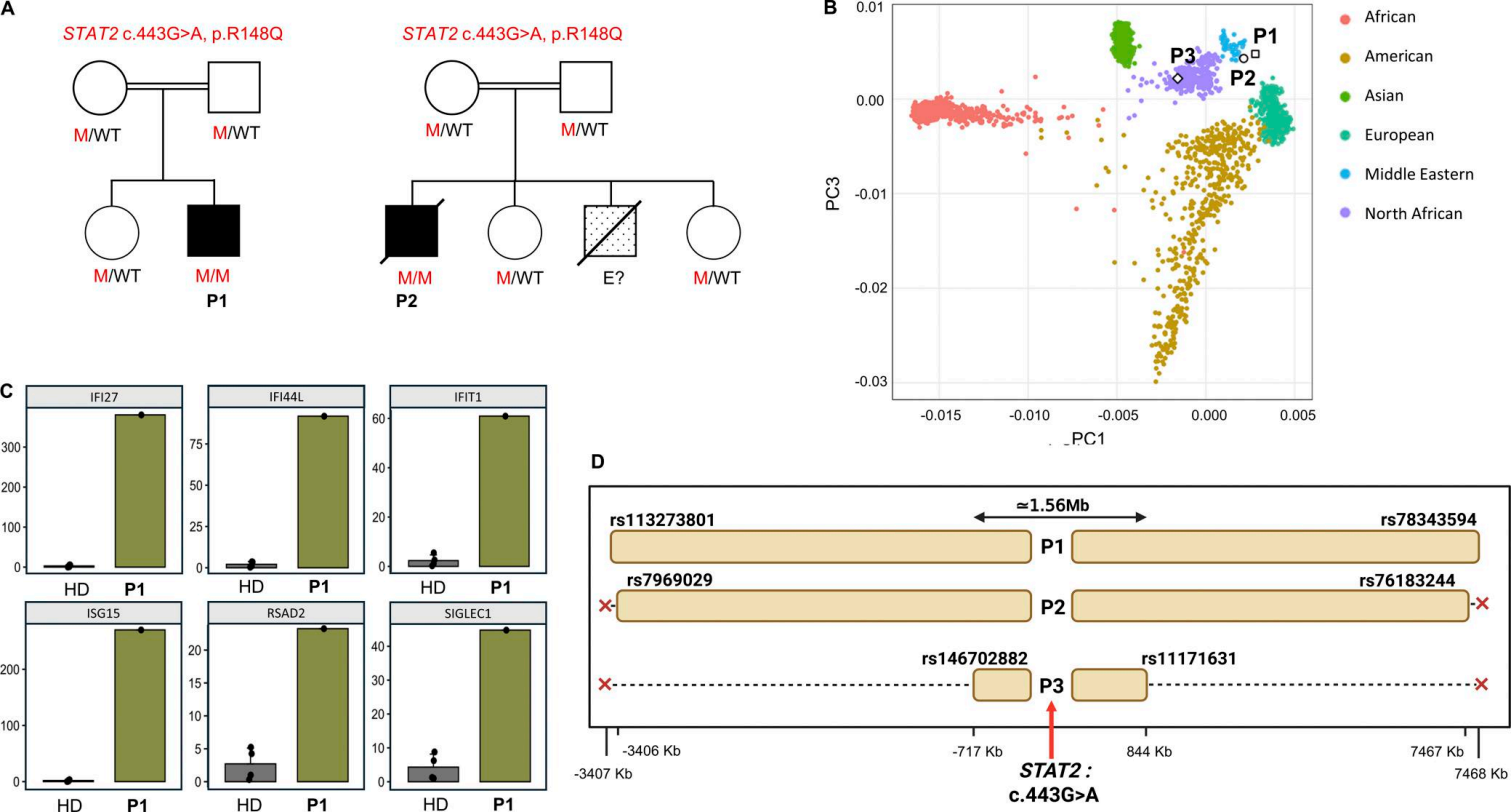
**Identification of biallelic *STAT2* p.R148Q variants in and their population genetic characterization. (A)** Familial segregation of the c.443G>A *STAT2* variant in P1 and P2 patients and their families. Double horizontal lines indicate consanguinity; a black square represents affected patients (P1, P2); a dotted square indicates a suspected individual with a phenotype similar to the patient but unknown genetic status (E?); a diagonal slash indicates a deceased individual; a circle represents a female and a square represents a male; M, mutant; WT, wild type. **(B)** Principal component analysis (PCA) of WES data of the patients (P1, P2, and P3), our in-house database, and samples from the 1,000 Genomes database. **(C)** Expression of *ISG* mRNA in whole blood collected from P1 (*n* = 1) and healthy donors (HD) (*n* = 4). **(D)** Shared haplotype around the c.443G>A *STAT2* variant of patients (P1, P2, and P3). The distance from the mutation is represented on the x-axis. Long continuous stretches of homozygosity were observed around the gene, consistent with its recessive mode of inheritance, and haplotypes were unambiguously derived from genotypes. The dbSNP reference numbers of the first and last unambiguous variants within the haplotype are reported for each carrier.

The male patient was born in 2022 from consanguineous parents of Iranian origin (Talesh, Gilan) after a normal vaginal delivery (Fig. 1, A–H and Fig. 2 A). He was vaccinated at birth with Bacillus Calmette-Guérin (BCG) vaccine on the left arm and oral polio vaccine (OPV) without any adverse effects. He first presented at 1.5 mo with draining cervical lymphadenitis on the right side that was negative for acid-fast bacilli and for culture of bacteria (Fig. 1 H). However, he was admitted and treated with antibiotics and was discharged after 14 days. Starting at 3 mo, he experienced seizures manifesting as staring spells, which improved after treatment with phenobarbital and levetiracetam. The electroencephalogram (EEG) study was unremarkable, but the computed tomography (CT) scan of brain revealed parenchymal hypodensity in left frontal lobe and asymmetric multifocal punctate and linear calcifications in bilateral basal ganglia, brain stem, and in posterior margin of affected area in left frontal lobe (Fig. 1 A). Magnetic resonance imaging (MRI) showed abnormal signal intensities, hypointense on T1W and T2W-FLAIR sequences in the left frontal lobe and basal ganglia, with marginal cortical hemorrhage. On diffusion-weighted imaging/apparent diffusion coefficient (DWI/AFC) map sequences, just marginal restriction mostly in hemorrhagic cortex was visualized, and no restriction on other affected regions was depicted, suggestive of focal brain edema, inflammation, and possible small vessel involvement and resultant marginal hemorrhage and encephalitis (Fig. 1 B). Since 3 mo, he was admitted three times with respiratory distress and hypoxia, along with diffuse lung opacities suggestive of pneumonia (Fig. 1 E). He received supplemental oxygen, antibiotics, and budesonide/salbutamol nebulization during admission. Provisional immunologic studies were uneventful. At 5 mo, he was admitted to the Pediatric Intensive Care Unit with respiratory distress, hypoxia (SpO_2_ 78% on room air), and irritability. He had significant anemia and a very high C-reactive protein level. Both the lung spiral CT scan and the chest x-ray revealed bilateral alveolar opacities consistent with bronchopneumonia; however, the spiral CT scan was not consistent with pulmonary alveolar proteinosis (PAP) (Fig. 1 F).

He received supplemental oxygen via an oxygen hood and was fed through a nasogastric tube. Broad-spectrum antibiotics (meropenem and vancomycin) were initiated. He began receiving noninvasive ventilation using a mask on the third day of his hospital stay due to a decline in his respiratory function. Bronchoalveolar lavage (BAL) performed on day 5 showed evidence of acute inflammation, showing many inflammatory cells, including mainly neutrophils and rare lymphoplasma cells, and macrophages in a clear background; however, the results were not characteristic for PAP. No pathogenic organisms were identified in the BAL fluid by culture. Methylprednisolone pulses (30 mg/kg for 3 consecutive days) followed by methylprednisolone (2 mg/kg/day during the admission tapered over 3 mo with oral prednisolone) were started. Additionally, following the confirmation of the *STAT2* variant (to be discussed below), ruxolitinib, a selective inhibitor of JAK1 and 2 (5 mg every 12 h), was initiated and continued (Fig. 1 H). Throughout his 35-day hospital stay, he got further supportive care, such as respiratory physical therapy and anticonvulsant medication. His SpO_2_ was steady at discharge (92–95%), but he still needed nasogastric (NG)-tube feeding because of lingering swallowing difficulties. During his 11 mo of follow-up at the outpatient clinic, he did not have any respiratory problems. Three months after starting ruxolitinib, a follow-up lung CT scan revealed modest peribronchial thickening (Fig. 1 G). At 17 mo, Denver II testing for developmental assessment showed abilities consistent with a developmental age of 16 mo. A follow-up brain MRI performed 3 mo after the initiation of therapy showed atrophy and encephalomalacia in the left frontal lobe and left basal ganglia in favor of past ischemic events. The last brain MRI performed at 17 mo showed no active lesion (Fig. 1 D). The patient is doing well at 36 mo with no complaints and is currently on ruxolitinib (5 mg every 12 h). Both parents and his older sister are healthy.

#### Patient 2 (P2) (Kindred B)

A male infant was the first child of consanguineous parents from Iran (Khalkhal, Ardabil). He was born in 2011 via vaginal delivery and received routine neonatal immunizations according to the national schedule (Fig. 2 A) without adverse effects of BCG and OPV vaccines. At 20 days of life, he developed progressive lymphadenopathy, initially in the inguinal region and subsequently involving the submandibular and cervical chains, with smaller nodes in both axillae. The inguinal nodes spontaneously suppurated and drained. Biopsy of an inguinal lymph node demonstrated fibroadipose tissue with necrosis and acuteinflammation. He received empirical antibiotics and was discharged without definitive therapy. At 2 years, the patient was admitted with decreased consciousness; a brain MRI was consistent with encephalitis. A PCR for herpes simplex virus on cerebrospinal fluid was negative. He improved after supportive care. At 3 years, two episodes of complex febrile seizures occurred; the EEG was abnormal, and he was managed with long-term phenobarbital therapy. The brain CT scan at that time showed bilateral cerebral calcifications in the basal ganglia. He subsequently developed intermittent skin ulcers located on the chin, periauricular area, and scalp managed with outpatient antibiotics. During the following years, he experienced recurrent respiratory symptoms and fever attributed to viral infections, but no etiologies were documented. At the last visit in 2019, he had poor school performance. At 8.5 years, he developed severe respiratory distress requiring hospitalization and passed away from respiratory failure. He had a younger brother who presented with generalized lymphadenopathies during the neonatal period and passed away at 3 mo due to progressive neurologic problems as refractory seizures and spasticity. Initial immunologic evaluations for both P1 and P2 were unremarkable, and no infectious organisms were isolated (Table S1); therefore, an autoinflammatory disorder was suspected.

### Genetic results and estimation of age to the most recent common ancestor

An inborn error of immunity (IEI) was suspected in both patients (P1 and P2), and whole-exome sequencing (WES) was performed. Ancestry was assessed by principal component analysis, and high homozygosity rates at 10.8% in P1 and 9.07% P2 confirmed parental consanguinity in kindreds A and B (Fig. 2 B). The same *STAT2* variant (c.443G>A, p.R148Q) was identified in both patients, who came from distinct families in the neighboring provinces of Gilan and Ardabil. No other genes underlying known IEIs had rare non-synonymous or copy number variants that could be related to the patient’s clinical phenotype. Sanger sequencing confirmed the variant in a homozygous state in P1 and P2 and the carrier status in their family members (Fig. 2 A). This pathogenic variant has already been reported in another patient from Morocco, hereafter referred to as P3 (10). Consistent with this initial report, ISG expression was markedly elevated in peripheral blood from P1 as compared to healthy donors, validating the functional consequence (Fig. 2 C). Analysis of WES data revealed that all three patients carrying the *STAT2* c.443G>A variant share a homozygous haplotype around the *STAT2* gene encompassing a 1.56-Mb region (Fig. 2 D). The shared homozygous region corresponds to 162 common single nucleotide variants. The Gamma approach estimated the age of the most recent common ancestor (MRCA) of these patients at ∼18.2 generations (95% confidence interval [CI]: 8–42.2 generations). Assuming a generation time of 27 years (16), the MRCA of these patients (P1, P2, and P3) with this variant would have lived about 491 years ago (95% CI: 216–1,139 years ago), indicating a relatively recent emergence of this founder variant within the background population. The nearly identical haplotype block sizes and genomic boundaries in P1 and P2 provide molecular evidence consistent with a more recent shared ancestor compared with P3.

### Review of the literature of patients with variants affecting negatively the STAT2 function

Seven (five published and two new) patients with homozygous rare STAT2 variants have been reviewed (9, 10, 11, 17) (Table 1). These patients originate from six unrelated kindreds from Iran, Morocco, Pakistan, Russia, and Turkey. Three homozygous missense variants have been reported: p.R148Q, p.R148W, and p.A219V. Despite underlying molecular distinctions, all three variants functionally disrupt USP18-mediated negative regulation, failing to terminate type I IFN signaling. All reported deleterious variants have been associated with upregulation of ISGs, demonstrated by quantitative PCR (qPCR) or NanoString assays. Affected patients developed early-onset multisystem inflammation (Table 1). Six patients presented within the first 6 wk of life, with a median onset at 4 wk (range: 15 days to 5 mo). Neurological manifestations were universal, including seizures (6/7, 86%). All patients demonstrated intracranial calcifications on neuroimaging, and additional findings included brain atrophy, encephalomalacia, white matter changes, and intracranial hemorrhage. All surviving patients had neurodevelopmental delay, except P1, who showed age-appropriate development at 36 mo (30 mo after initiation of JAK inhibitor).

**Table 1.** Clinical and genetic features of patients harboring STAT2 variants with negative regulation

Family	Patient	Origin	Homozygous variant	Age at onset	Neurological features	Others	ISG signature	JAK inhibitor response	Outcome	Study
F1	P1	Iran	c.443G>A p.R148Q	6 wk	Seizure Intracranial calcifications Hemorrhage Atrophy encephalomalacia	Draining cervical adenitis Respiratory failure	Upregulated	Ruxolitinib, dramatic response to 5 mg, bid	Favorable developmental and respirator function at 24 mo 3 years old at 2025	Current
F2	P2	Iran	c.443G>A p.R148Q	20 days	Seizure Intracranial calcification Encephalitis Abnormal EEG	Draining adenitis Fever Respiratory failure	NA	No	Died at 8.5 years with influenza-induced respiratory failure	Current
F3	P3	Morocco	c.443G>A p.R148Q	15 days	Seizures Intracranial calcifications	Adenitis (*P. aeruginosa* and *K. pneumoniae*) Fever PAP Respiratory failure skin ulcerations Cardiomegaly	Upregulated	No	Died at 5 mo from respiratory failure	Gruber et al. (2020) (10)
F4	P4	Pakistan	c.442C>T p.R148W	Neonatal	Seizure Intracranial calcification Hemorrhage White matter changes Neurodevelopmental delay	Thrombocytopenia Recurrent HLH-like episodes Thrombotic microangiopathy Acute kidney injury Proteinuria	Upregulated	Ruxolitinib 2.5 mg bid, clinical stabilization and improved ISG score	Died in immediate post-HSCT period with sepsis (20 mo)	Duncan et al. (2019) (9)
	P5	Pakistan	c.442C>T p.R148W	3 wk	Recurrent apnea Intracranial calcification Hemorrhage White matter changes Cerebellar hypoplasia Brainstem atrophy Abnormal EEG	Preterm birth	Upregulated	Ruxolitinib 1 mg bid, partial response, improved ISG score but persistent neurodevelopmental abnormalities	Diet at 3 mo with severe neurologic impairment	Duncan et al. (2019) (9)
F5	P6	Russia	c.442C>T p.R148W	Neonatal	Seizures Intracranial calcifications Neurodevelopmental delay	Adenitis after BCG Fever Interstitial pneumonia and bronchiolitis	NA	Tofacitinib, 20 mg per day with clinical and picture improvements	5 years old in 2021	Kozlova et al. (2021) (17)
F6	P7	Turkey	c.656C>T p.A219V	5 mo	Intracranial calcifications Cerebral atrophy Delayed motor and speech Spastic paraparesis Stroke-like episodes of hemiparesis Pontine hemorrhage dystonia	Fever Abnormal liver function	Upregulated Tested by qPCR and NanoString assays	No	Severe neurologic sequelae at 20 years	Zhu et al. (2023) (11)

HLH, hemophagocytic lymphohistiocytosis; mo, months; wk, week.

Four patients (P1, P2, P3, and P6) experienced draining lymphadenitis in the cervical and inguinal regions. Adenitis in P3 was positive for *Pseudomonas aeruginosa *and *Klebsiella pneumoniae,* and adenitis in P6 was referred after BCG vaccine. These four patients experienced recurrent fever episodes as well. Severe respiratory involvement occurred in three patients (P1, P2, and P3) carrying the same p.R148Q variant. All developed diffuse lung opacities progressing to respiratory failure requiring intensive care. BAL in P1 revealed acute neutrophilic inflammation. P6, carrying the p.R148W variant, also had interstitial pneumonia diagnosed by lung biopsy. Regarding extrapulmonary manifestations, additional systemic features included thrombocytopenia and hemophagocytic lymphohistiocytosis-like episodes in P4, as well as chronic skin ulcerations in P2 and P3. Clinical outcomes varied, but mortality was high (57%), primarily due to respiratory failure or severe neurological complications. Notably, mortality might be even higher in the absence of targeted intervention. JAK inhibitor therapy with ruxolitinib was used in three patients with variable responses. P1 responded dramatically to ruxolitinib 5 mg twice daily, with normalization of respiratory function and favorable neurodevelopment. P4 received 2.5 mg twice daily with neurologic improvement and suppression of ISG signature; however, he died from post-HSCT sepsis at 20 mo. P5 received 1 mg twice daily with biochemical improvement but persistent neurological impairment and died at 3 mo. A fourth patient (P6) received high-dose tofacitinib (20 mg/day) with clinical and chest CT improvements (17).

## Discussion

We report here two new patients from Iran carrying the *STAT2* R148Q variant. Since its first description in 2019, this IEI has been reported in patients from several countries, including Morocco, Pakistan, Russia, and Turkey. These *STAT2* variants are inherited in an AR mode (9, 10, 11, 17). This exceptional recessive pattern for STAT2 likely reflects a specific molecular mechanism, whereby biallelic variants are required to completely disrupt USP18-mediated negative feedback of type I IFN signaling. The estimated age of the MRCA (∼491 years ago; 95% CI, 216–1,139 years) suggests that the *STAT2* c.443G>A, p.R148Q variant emerged in the early 16th century CE, during the Safavid dynasty in Iran and the Saadi dynasty in Morocco. Despite their political distinctions, both were part of a broader Islamic network linked by trade, pilgrimage, and scholarly interchange across the Middle East and North Africa. These transregional interactions, supported by genomic evidence of continuous gene flow across these regions (18, 19), may have facilitated the dissemination of ancestral alleles. The persistence of this variant in both populations may then reflect local endogamy and genetic drift reinforcing a common founder effect. The presence of nearly identical haplotype blocks (in size and position) of P1 and P2 is proof of an MRCA compared to P3. In addition, determining of the minor allele frequency of this variant in specific populations may support consideration of population-based carrier screening may be considered in regions with high consanguinity, particularly where this founder mutation has been observed (20, 21).

USP18 normally binds to STAT2 and IFNAR2, promoting the displacement of JAK1 from IFNAR2, and thereby terminating IFN responses. The clinical phenotype of STAT2 LOF/regulation closely resembles and is effectively is a phenocopy of AR partial USP18 deficiency, highlighting a shared pathophysiology in which impaired USP18-mediated negative feedback results in unregulated type I IFN signaling and severe interferonopathy (22, 23). In addition, STAT2 R148LOF/regulation is a phenocopy of complete ISG15 deficiency (24, 25, 26). While the resulting inflammation is sterile, the clinical presentation suggests that infectious triggers likely precipitate the disease. The biological requirement for an initial IFN stimulus to induce USP18, combined with the variability in age of onset and the association of disease flares with infectious symptoms, supports the hypothesis that an external “second hit” sets off the unregulated signaling loop. This feature distinguishes STAT2 R148 variant from STAT2 LOF deficiency, in which viral exposure typically precipitates disease manifestations (1, 2, 3, 4, 5, 6, 7, 8, 9, 10, 11). The neurotropism of the inflammatory process, evidenced by universal intracranial calcifications and high rates of seizures, likely reflects the particular vulnerability of the developing nervous system to chronic IFN exposure (27, 28). Our findings add to the growing body of evidence that early and sustained type I IFN activation in infancy contributes directly to neuroinflammation and long-term neurologic sequelae.

This study defines the clinical spectrum of STAT2 R148 disease and illustrates the potential for targeted therapy using JAK inhibitors. Treatment with JAK inhibitor has already reported in complete USP18 deficiency and other type I interferonopathies, followed by a good clinical response (29, 30, 31, 32, 33). The variable response to JAK inhibitor therapy of this small number of reported STAT2 R148 patients underscores the importance of early intervention and appropriate dosing strategies. P1 exhibited a dramatic and sustained response to ruxolitinib 5 mg twice daily when initiated early in the disease course. In contrast, P4, treated at 2.5 mg twice daily, showed partial improvement before dying from post-HSCT sepsis, and P5, treated with 1 mg twice daily, had biochemical improvement but persistent severe neurological impairment and died at 3 mo. Moreover, P6 showed clinical improvement with high-dose tofacitinib (20 mg/day), indicating that high-intensity JAK inhibition may be beneficial in select cases. Poor outcomes in untreated patients (P2, P3, and P7) highlight the devastating natural history of this condition and the need for prompt recognition and therapeutic intervention (9, 10, 11). Combined therapy is necessary in these patients, as described for other type I interferonopathies (33). The limited number of STAT2-deficient patients treated with JAK inhibitors remains a constraint. The incorporation of JAK inhibitors as part of treatment regimens is encouraging. However, prognosis remains guarded, with significant mortality. Timely initiation of JAK inhibitors, with dosage titrated to clinical response, may improve outcomes. JAK inhibitors were also used as therapy in AR STAT2 LOF, serving as targeted anti-inflammatory drugs to control hyperinflammation (1). Further studies are needed to refine dosing strategies, clarify optimal timing of therapy, and evaluate long-term safety in infants and young children. A coordinated international registry would greatly facilitate these efforts and improve clinical management of this rare but potentially treatable interferonopathy.

## Materials and methods

### Human subjects

Written informed consent was obtained from the patients’ parents in accordance with local regulations and with Institutional Review Board (IRB) approval of the Children’s Medical Center affiliated with the Tehran University of Medical Sciences in Iran. Ethylenediaminetetraacetic acid whole-blood samples from patients and relatives were collected.

### Sequencing

Genomic DNA was extracted from peripheral whole blood using the MG Blood Genomic DNA extraction Miniprep (CancerRop) according to the manufacturer’s instruction. Clinical WES was performed on patient samples (3 µg), as reported previously (26). Exome capture was performed with the SureSelect Human All Exon 50 Mb kit (Agilent Technologies). Paired-end sequencing was performed on a HiSeq 2000 (Illumina), generating 100-base reads. Bidirectional sequence reads were assembled and aligned to the human genome build GRCh38. Downstream processing and variant calling were performed with the Genome Analysis Toolkit, SAMtools, and Picard. Substitution and InDel calls were made with GATK Unified Genotyper. All variants were annotated using an annotation software system that was developed in-house. The status of variants has been documented by Sanger sequencing The region of interest in exon 5 was amplified using the following primers: STAT2-5′-ACCATGGAAAGGACTCAGGGA-3′ and STAT2-5-5′-TGTTAGGCTGAACGCTGTCAA-3′.

### Homozygosity mapping and dating of mutation

We utilized PLINK (https://www.cog-genomics.org/plink/) to extract the runs of homozygosity (ROHs) from the ES data after converting the BAM file to Variant Call Format. ROH was found using PLINK’s ROH algorithm. The PLINK default settings are suitable for detecting large parts of ROH on dense genotyping platforms and remained constant throughout the investigation (21). Dating of the variation was performed using previously published methods for rare mutations in small sample sets with dense marker data (https://github.com/bahlolab/DatingRareMutations) (34, 35). The model assumes a correlated genealogy, suggesting that subsets of individuals are likely to share common ancestry before the MRCA, given that individuals rarely exhibit independent recombinational histories (35).

### RNA isolation and qPCR

Whole-blood samples were collected into PAXGene Blood RNA tubes (762165; Qiagen), and RNA was isolated as per the manufacturer’s instructions (762174; Qiagen). cDNA was generated by reverse transcription (High-Capacity cDNA Reverse Transcription Kit, 4374967; Thermo Fisher Scientific). The expression of six standard ISGs (*IFI27*, *IFI44L*, *IFIT1*, *ISG15*, *RSAD2*, and *SIGLEC1*) and a housekeeper gene (*GAPDH*) was analyzed by TaqMan quantitative real-time PCR (TaqMan Universal Master Mix II with UNG) on QuantStudio 6 Pro Real-Time PCR System (Thermo Fisher Scientific) as previously described (10). The delta–delta Ct method was used to derive relative expression values.

### Online supplemental material

Table S1 shows laboratory findings of patients P1 and P2.

## Ethics approval

Patient recruitment was overseen by the Children’s Medical Center Institutional Ethics Committee, affiliated with Tehran University of Medical Sciences.

## Consent to participate

Written informed consent for participation was obtained from the patient’s family. The analysis of patients’ samples was approved by the French Ethics Committee “Comité de Protection des Personnes,” the French National Agency for Medicine and Health Product Safety, the Institut National de la Santé et de la Recherche Médicale, in France, and the Rockefeller University IRB in New York.

## Consent for publication

Written informed consent for publication of the patient’s clinical data was provided by the families.

## Supplementary Material

Table S1 shows laboratory findings of patients P1 and P2.

## Data Availability

The genetic data for the patients in this study have been submitted to the ClinVar database under the accession link: https://www.ncbi.nlm.nih.gov/clinvar/variation/VCV000907069.5.
