# Supplementary material for: STAT2 R148 variant: A 16th-century founder mutation and clinical response to high-dose JAK inhibitor therapy
Source: J Hum Immun. 2026 May 27;2(4):e20260001. doi: 10.70962/jhi.20260001 (PMC13215009; doi:10.70962/jhi.20260001)
Supplement: Table S1 — shows laboratory findings of patients P1 and P2. [file jhi_20260001_tables1.docx]

|  | **P1 at 6 months** | **Normal** | **P2 at 5 years** | **Normal** |
| --- | --- | --- | --- | --- |
| **WBC (cells/μL)** | 9360 | 4400-9500 | 11470 | 5800-13600 |
| **Hb (mg/dL)** | 8.8 | 11-16 | 10 | 9.4-12.5 |
| **Platelets (×10^9^/L)** | 339 | >150 | 343 | >150 |
| **Neutrophils (cells/μL)** | 3830 | 2000-7000 | 6220 | 1590-6640 |
| **Lymphocytes (cells/μL)** | 4940 | 1900-3700 | 4900 | 1930-6110 |
| **CD3 (cells/μL)** | 3062 | 1400-3700 | 2400 | 1200-2600 |
| **CD4 (cells/μL)** | 2667 | 700-2200 | 1602 | 650-1500 |
| **CD8 (cells/μL)** | 494 | 490-1300 | 800 | 370-810 |
| **CD19 (cells/μL)** | 1531 | 390-1400 | 962 | 270-860 |
| **CD56 (cells/μL)** | 247 | 120-520 | 1520 | 100-480 |
| **IgG (mg/dL)** | 584 | 215-704 | 390 | 270-1100 |
| **IgM (mg/dL)** | 169 | 31-132 | 85 | 47-170 |
| **IgA (mg/dL)** | 51 | 8.1-68 | 53 | 12-90 |
| **Anti-tetanus IgG (IU/mL)** | ND | >0.1 protective | 5 | >0.1 protective |
| **Anti-diphtheria IgG (IU/mL)** | ND | >0.1 protective | 2 | >0.1 protective |
| **ESR-1hr (mm/hr)** | 27 | 0-10 | ND | 0-10 |
| **CRP (mg/L)** | 76 | >10 positive | ND | >10 positive |
| **Dihydrorhodamine (DHR) test** | 130 | >30 normal | 94 | >30 normal |
| **Pro-calcitonin (ng/ml)** | 7.6 | 2-10 systemic inflammation | ND | 2-10 systemic inflammation |
| **Ferritin (ng/ml)** | 119 | <400 | ND | <400 |
| **EBV viral load** | Negative | Negative | ND |  |
| **CMV viral load** | Negative | Negative | ND |  |
| **COVID-19 PCR** | Negative |  | ND |  |
| **HIV PCR** | Negative |  | Negative |  |
| **Blood culture** | Negative |  | ND |  |
| **BAL culture** | Negative |  | ND |  |
| **BAL MTB PCR** | Negative |  | ND |  |
| **BAL CMV PCR** | Negative |  | ND |  |

**Supplementary Table 1:** Laboratory findings of patients P1 and P2

BAL, bronchoalveolar lavage; CMV, cytomegalovirus; EBV, Epstein Barr virus; HIV, human immunodeficiency virus; MTB, *Mycobacterium tuberculosis* ; ND, not determined
